# Evaluation of the Tribological Behavior of Materials Used for the Production of Orthodontic Devices in 3D DLP Printing Technology, Due to Oral Cavity Environmental Factors

**DOI:** 10.3390/ma18020301

**Published:** 2025-01-10

**Authors:** Andrzej Snarski-Adamski, Daniel Pieniak, Zbigniew Krzysiak, Marcel Firlej, František Brumerčík

**Affiliations:** 1 Department of Mechanical Engineering and Automation, University of Life Sciences in Lublin, 20-612 Lublin, Poland; 2 Łukasiewicz Research Network-Institute for Sustainable Technologies, 26-600 Radom, Poland; daniel.pieniak@itee.lukasiewicz.gov.pl; 3 Department of Orthodontics and Facial Malformations, Poznan University of Medical Sciences, 60-812 Poznań, Poland; 4 Department of Design and Machine Elements, Faculty of Mechanical Engineering, University of Žilina, 010 26 Žilina, Slovakia; frantisek.brumercik@fstroj.uniza.sk

**Keywords:** orthodontic devices, 3D DLP print, polymer materials, oral environment, wear

## Abstract

This study evaluated the effect of oral cavity environmental factors on the friction and wear of materials used in 3D-printed orthodontic devices. Commercial materials GR-10 (Pro3Dure) and NextDent SG (NextDent) were examined, with samples produced using ASIGA UV MAX and Phrozen Shuffle Lite 3D printers. Our tests included measurements of hardness, stiffness, elastic modulus, cyclic loading, scratch resistance, and tribological assessments in oscillatory motion. Surface analyses were conducted using scanning electron microscopy with an energy-dispersive spectroscopy analyzer. The results showed that NextDent SG exhibited higher hardness and modulus of elasticity, while GR-10 demonstrated better scratch resistance. Despite similar friction coefficients, significant variations in wear were observed under different environmental conditions, highlighting the importance of considering these factors in the performance of orthodontic materials.

## 1. Introduction

Three-dimensional (3D) printing technology is revolutionizing dentistry, particularly through additive manufacturing methods like 3D digital light processing (DLP) printing. This technology enables the precise layer-by-layer fabrication of complex dental devices such as orthodontic splints [1], as well as models, surgical instruments, and prostheses [2,3,4,5]. Being more cost-effective than subtractive methods, in terms of machinery and material costs [6,7], it is increasingly adopted by dentists [8].

Polymer materials play a crucial role in orthodontics, due to their versatility and compatibility with the oral environment. They constitute a significant portion of medical devices [9,10], including volumetric elements and coatings [11,12]. Vat-polymerization technologies—encompassing stereolithography (SLA), DLP, and liquid crystal display-based (LCD) printers (also known as daylight polymer printing, DPP)—are widely used in dental manufacturing [13,14,15]. DLP technology offers excellent dimensional accuracy from CAD models and produces elements with low surface roughness [16], making it ideal for creating precise orthodontic products.

The rapid adoption of 3D DLP printing in orthodontics underscores the need for new materials specifically engineered for this technology. Traditional materials may lack adequate wear resistance, mechanical strength, or biocompatibility when exposed to the complex conditions of the oral cavity, which includes mechanical forces, thermal fluctuations, moisture, and chemical interactions. Developing advanced materials with enhanced tribological properties—such as improved friction and wear resistance—is essential for the longevity and effectiveness of orthodontic devices. This need has been heightened by the growing demand for personalized dental care, where devices are customized to individual patient anatomies.

Optimizing orthodontic device performance requires establishing a quantitative relationship between tribological behavior and 3D DLP printing technology. Materials’ tribological properties, like friction coefficient and wear rate, are influenced by surface characteristics affected by DLP printing parameters. Factors such as layer thickness, curing time, light intensity, and printing orientation can significantly alter microstructure and surface topography [17]. For example, finer layer thicknesses reduce surface roughness, leading to lower friction coefficients, while optimal curing ensures adequate cross-linking for better wear resistance [18]. Precisely controlling these parameters enables the engineering of materials with tailored properties for specific clinical needs.

**Figure 1:**
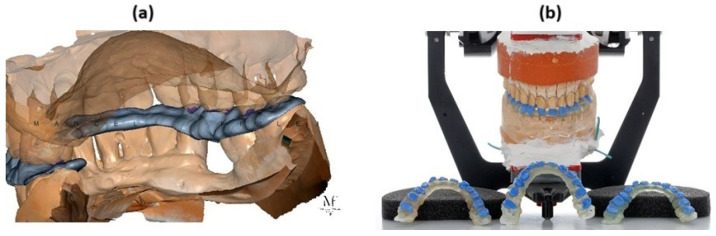
CAD model of the so-called thin orthodontic splint: (**a**) printed splint and layers; (**b**) in the places of cuspation of opposing teeth, which help to establish the target relationship of the maxilla and mandible.

Physiological factors in the oral cavity—such as biomechanical forces, temperature, and humidity—impact the friction and wear of dental materials working within biotribological systems. Wear resistance can be influenced by nutritional habits, environmental conditions, and malocclusion. Orthodontic splints, for instance, may suffer abrasive damage due to contact with teeth. Thin splints are a novel solution used to register the bite and stabilize the position of the maxilla and mandible, often enhanced with composite materials (Figure 1). They must withstand bite forces and maintain their designed profile over extended periods.

Experimental observations show that polymer occlusal devices can exhibit deformation and wear at contact points with teeth, correlated with maximum muscle activity during sleep and involuntary teeth grinding in stressful situations. Sharp irregularities on tooth edges, resulting from enamel chipping or inaccurate cavity fillings, can cause tribological damage to these devices.

Recognizing that biomechanical forces, temperature, and humidity all affect friction and wear, this study aimed to assess the impact of isolated oral cavity environmental factors on the friction and wear of materials used for orthodontic devices fabricated using 3D DLP printing technology. By applying J.S. Mill’s rule of eliminative induction to isolate specific variables, we sought to establish a clear quantitative relationship between tribological behavior and environmental conditions. For example, it can be assumed that in addition to mechanical forces, two additional factors—thermal and moisture—affect the friction and wear of the surfaces of the materials under study in the first case; in the second case, only one of them: the absence of one of the circumstances in this case may be the result or cause of friction and wear phenomena [19]. In addition to assessing the functional properties of the surface layer of the tested materials, the main goal of this work was to assess the impact of isolated oral cavity environmental factors on the friction and wear of materials used for orthodontic devices.

## 2. Materials and Methods

In this study, we simulated specific oral conditions by varying humidity and temperature to isolate their effects on the friction and wear of orthodontic materials. Other influential factors, such as saliva chemistry and the presence of food particles, were not included in the current experimental setup, to maintain a controlled investigation of these isolated variables.

This study examined commercial materials designed for 3D-printed dental appliances. GR-10 guide is a material manufactured by Pro3Dure (Iserlohn, Germany), and samples from this material were made on the ASIGA UV MAX printer (light source 385 nm). NextDent SG material is manufactured by NextDent (Soesterberg, Netherlands), and samples from this material were made on the Phrozen Shuffle Lite 3D printer. Both printers use DLP (digital light processing) technology. Phrozen Shuffle Lite (PSL) is equipped with a 5.5-inch LCD screen with an XY range of 2560 dpi × 1440 dpi and a resolution of 47 µm. ASIGA UV MAX (ASM) features a 4.5-inch LCD screen with an XY range of 1919 × 1081 dpi and a resolution of 62 µm. The samples were cylindrical discs 30 mm in diameter and 6 mm high. All prints were rinsed in 99.9% isopropyl alcohol for 6 min to remove any residual uncured resin and then light-cured in an Anycubic all-in-one Wash & Cure machine (HONGKONG ANYCUBIC TECHNOLOGY CO., London, UK) for 4 min. The samples were then sanded and polished with abrasive discs of increasing granularity, i.e., P600, P1200, and P2400, and they were polished with a cloth-covered disc with a diamond suspension (1 µm) with a Saphir 550 single-disc automatic sander and polisher (ATM Gmbh, Mammelzen, Germany).

The surface roughness of the samples after polishing was assessed using a Taylor Hobson Talysurf CCI (CCI, Taylor Hobson Ltd., Leicester, UK) optical profilometer. The principle of operation of this device is based on scanning white light interferometry. The device allows for the generation of three-dimensional surface images. The following surface parameters were determined in accordance with ISO 25178 [20]: Sq—root mean square height of the 3D profile; Sp—height of the highest peak of the 3D profile; Sv—depth of the deepest valley of the 3D profile; Sz—maximum height of the 3D profile; Sa—arithmetic mean height of the 3D profile; Ssk—skewness; and Sku—kurtosis.

1. GR-10 Guide Resin with ASIGA UV Max Printer
  - Material: GR-10 guide, manufactured by Pro3dure medical GmbH.
  - Printer: ASIGA UV Max (ASM) (Asiga, Alexandria, NSW, Australia).
    - Technology: digital light processing (DLP)
    - Light Source: 385 nm
    - LCD Screen: 4.5-inch with an XY resolution of 1919 × 1081 dpi
    Pixel Size (Resolution): 62 μm
2. NextDent SG Resin with Phrozen Shuffle Lite Printer
  - Material: NextDent SG, manufactured by NextDent.
  - Printer: Phrozen Shuffle Lite (PSL) (Phrozen, Hsinchu City, Taiwan).
    - Technology: digital light processing (DLP)
    - LCD Screen: 5.5-inch with an XY resolution of 2560 × 1440 dpi
    - Pixel Size (Resolution): 47 μm

### 2.1. Specimen Fabrication and Printing Parameters

#### 2.1.1. GR-10 Resin with ASIGA UV Max Printer

Specimens made from GR-10 resin were fabricated using the ASIGA UV Max printer with parameters specified by the manufacturer:

- General Settings:
  - Material Profile Name: GR-10 clear-transparent—385
  - Heater Temperature: 30 °C
  - Heater Enabled: Yes
- Burn-In Layers:
  - Number of Layers: 1
  - Burn-In Exposure: 0.5 mm
- XY Scale Adjustment:
  - Scale Factor: 1.0085 (to compensate for resin shrinkage)

Support Settings:

- Leveling Enabled: Yes
  - Leveling Height: 2.0 mm
- Tallest Feature Detection: Disabled
- Slope Angle: 20 °
- Side Feature Size: 2.0 mm
- Support Spacing: 3.0 mm
- Support Strength: 40
- Intersupport Connection: Enabled
- Contact Point Diameter: 0.5 mm
- Overshoot Length: 1.0 mm
- Maximum Support Width: 2.3 mm
- Side Faces on Supports: 8
- Aspect Ratio: 3.0

#### 2.1.2. NextDent SG Resin with Phrozen Shuffle Lite Printer

Specimens made from NextDent SG resin were fabricated using the Phrozen Shuffle Lite printer with parameters provided by the resin manufacturer for open system printers:

- General Settings:
  - Layer Height: 0.05 mm
  - Retract Speed: 100 mm/min
  - Light-Off Delay: 3 s
- Bottom Layers (First 6 Layers):
  - Exposure Time: 35 s
  - Lift Height: 8 mm
  - Lift Speed: 50 mm/min
- Normal Layers:
  - Exposure Time: 3.5 s
  - Lift Height: 7 mm
  - Lift Speed: 50 mm/min

### 2.2. Justification of Parameters

- Manufacturer Recommendations: The printing parameters for both resins were selected based on the manufacturers’ guidelines, to ensure optimal curing and mechanical properties.
  - For GR-10 Resin: The manufacturer Pro3dure provides parameters specifically for the ASIGA UV Max printer.
  - For NextDent SG Resin: NextDent supplies parameters suitable for open 3D printing systems like the Phrozen Shuffle Lite.
- Reproducibility and Accuracy: Detailed printing parameters are provided, to ensure that the study can be accurately replicated by other researchers and to maintain consistency in specimen fabrication.

### 2.3. Indentation Properties

A Micro Combi Tester universal microhardness tester (MCT, Anton Paar GmbH, Ostfildern, Germany) was used to measure the mechanical and elastic surface parameters of the tested samples. The course of the test load of the Vickers indenter included three phases. Indentation hardness $H_{IT}$ was determined as the ratio of the highest normal force loading the indenter Pmax to the contact area of the indenter under maximum load A, according to Formula (1) [21]:

(1) $$H_{IT}=\frac{P_{max}}{A}.$$

Stiffness S was determined from relationship (2) [21]:

(2) $$S=\frac{dP}{dh}=\beta \cdot \left(\frac{2}{\sqrt{\pi }}\right)\cdot E\cdot \sqrt{A}.$$

The parameter β, in the case of any symmetrical indenter, is assumed to be equal to 1. For the Vickers indenter, the corrected value of the parameter β was 1.0055 [21].

(3) $$A=F\left(h_{c}\right)=24.54h_{c}^{2}+C_{1}h_{c}^{1}+C_{2}h_{c}^{\frac{1}{2}}+C_{3}h_{c}^{\frac{1}{4}}+C_{4}h_{c}^{\frac{1}{8}}+\cdots +C_{n}h_{c}^{\frac{1}{2n}},$$

In Equation (3), the calculations are based on the constant $C_{n}$, which expresses the indenter geometry. The method of determining the constant $C_{n}$ is described in [22]. Based on the stiffness S calculated from Equation (2) and the penetration path of the indenter corresponding to the elastic deformations of the test surface, $h_{c}$, the indentation modulus of elasticity EIT was calculated according to Equation (4):

(4) $$E_{\mathrm{IT}}=\frac{\sqrt{\pi }S}{2\beta \sqrt{A\cdot h_{c}}},$$

Then, 100 indentation cycles were performed. Cyclic tests were conducted, using the function describing the shape of the load–time curve in the “Quadratic Loading” cycle. The following cycle parameters were assumed: max load 1 N, unload to 0.05 N, time to max load 1.0 s, time to unload 1.0 s, pause 0.1 s.

### 2.4. Scratch Test

**Figure 2:**
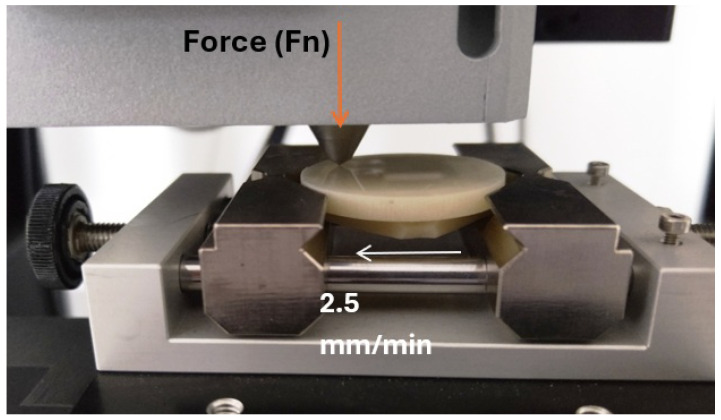
Scratch test on the MST device.

**Figure 3:**
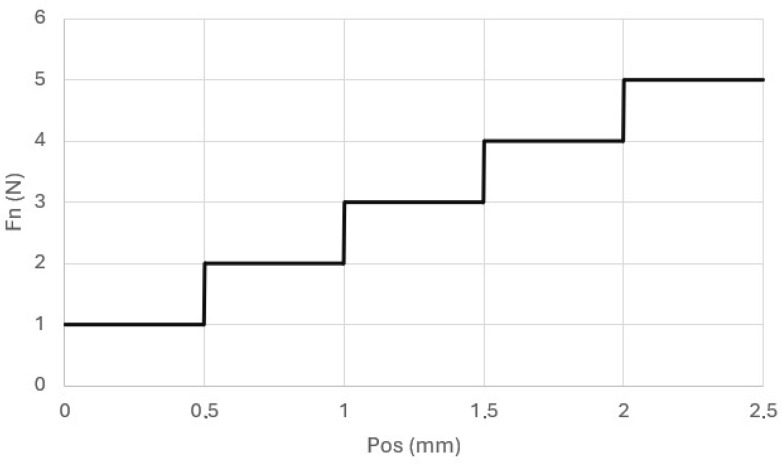
Normal force course in the scratch test.

In addition, we carried out scratch testing. The samples were tested on a Micro Scratch Tester (MST, Anton Paar, GmbH, Ostfildern, Germany). One of the surfaces of the samples was processed on a laboratory grinder-polisher. Scratches were made on this surface, using a Rockwell indenter (diamond cone with a rounding radius of 100 µm) (Figure 2). The test load, whose vector was perpendicular to the tested surface, was stepwise variable and increased by 1N in subsequent stages (Figure 3). The scratch test was conducted at a speed of 2.5 mm/min. The total scratch length was 2.5 mm. The shape and geometric dimensions of the scratch were assessed microscopically. The microscope was an integral part of the MST device.

### 2.5. Sliding Friction

**Figure 4:**
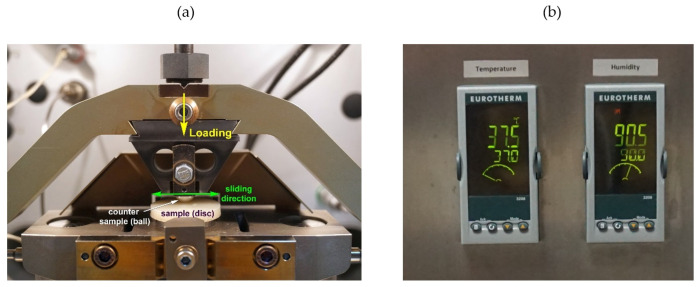

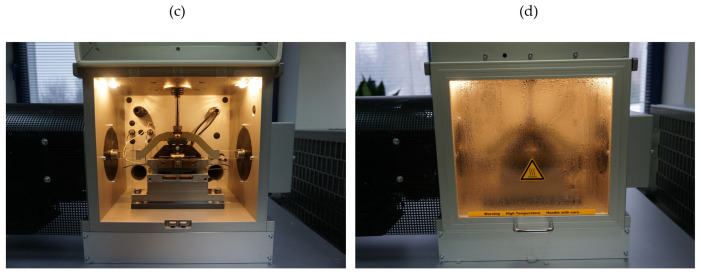
Tribological properties tests: (**a**) ball on disc test setup; (**b**) setting environmental parameters close to physiological; (**c**) environmental chamber during testing at 30% humidity (**d**) and 90% humidity.

Tribological tests were conducted in oscillatory motion (Figure 4a) on the SRV 4 tester (Optimol Instruments Prüftechnik GmbH, Munich, Germany). The SRV tester is equipped with a climatic chamber (Figure 4b,c), allowing the maintenance of a temperature of approx. 37 °C and humidity up to 90% (Figure 4d). Cylindrical discs made of 3D printed materials were used as samples, while the counter-samples were beads with a diameter of Ø 10 mm. The beads were made of steel (100Cr6). For each environmental condition, three tests were conducted.

## 3. Results

### 3.1. Surface Topography

**Figure 5:**
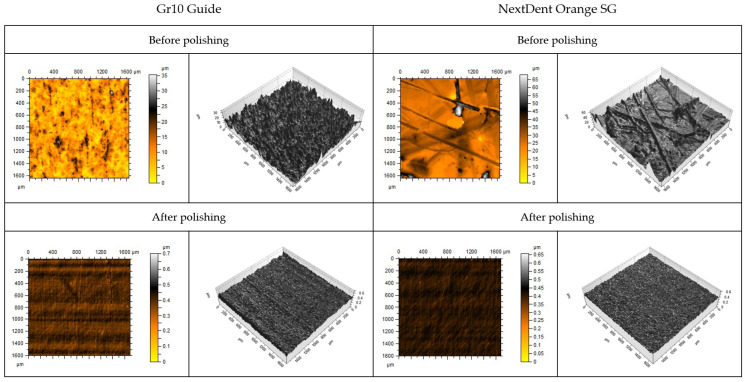
Surface topography of samples after printing and after grinding and polishing.

Figure 5 illustrates the profilometric study results, highlighting significant differences in surface topography between the materials. The profilograms show the surface condition of selected samples on a 1.6 mm × 1.6 mm surface after printing and after grinding and polishing. Clear differences between the materials are visible. The unground samples differed in surface topography. Larger irregularities were observed on the surface of the NextDent SG material samples. This relationship was also confirmed by the results presented in Table 1. The Sv and Sp of the NextDent SG material were significantly higher. The susceptibility of both materials to grinding and polishing underscores their potential for practical applications in dental prosthetics. The surface quality after abrasive treatment was significantly higher. Similar Sa and Sz values were achieved, which enabled comparative tribological studies.

### 3.2. Indentation Properties

**Figure 6:**
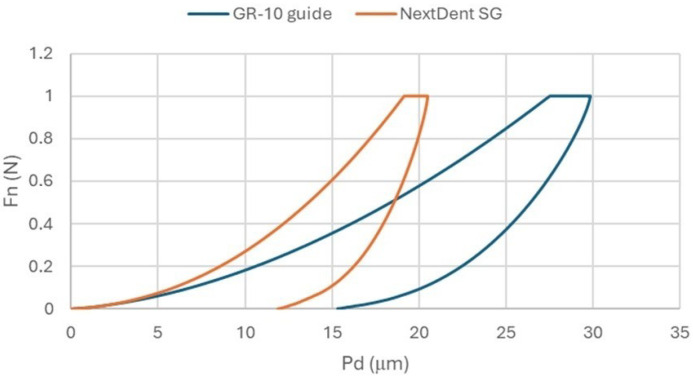
Mean of characteristics, normal load (Fn)-penetration depth (Pd).

Figure 6 presents the averaged curves of the normal force (Fn)-penetration depth (Pd) of the Vickers indenter into the surfaces of the tested materials. The study revealed notable differences in surface behavior under concentrated loads, which are critical for understanding material performance. The average penetration depth of the indenter under a load of 1N was approx. 20 µm for the NextDent SG material and approx. 30 µm for the GR-10 guide material. Such behavior under concentrated loads also translated into hardness and modulus of elasticity (Table 2). The average indentation hardness of the NextDent SG material was more than twice that of the GR-10 guide, as was the modulus of elasticity. Stiffness was less varied.

**Figure 7:**
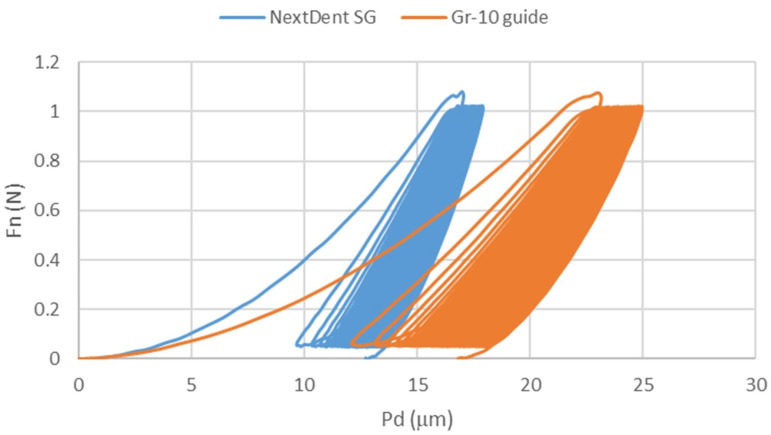
Characteristics, normal load (Fn)-penetration depth (Pd) for 100 measurements.

**Figure 8:**
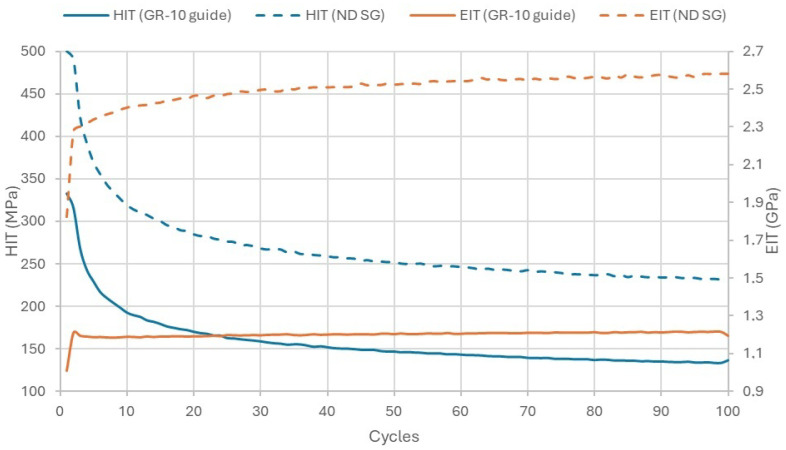
Hardness (HIT) and surface modulus of elasticity (EIT) in subsequent indentation cycles.

In the cyclic indentation test, Fn-Pd curves were obtained in subsequent cycles (Figure 7). Similar to the single-cycle test, the curves of the tested materials did not overlap, and the GR-10 guide was characterized by greater susceptibility. Changes in hardness (HIT) and modulus of elasticity (EIT) were observed in subsequent cycles (Figure 8). Hardness decreased in subsequent cycles, fastest at the beginning, and after about thirty cycles the intensity of the hardness decreased. The modulus of elasticity increased rapidly in the first few cycles, then stabilized, maintaining a linear trend slightly inclined towards the increasing modulus of elasticity.

### 3.3. Scratch Test Results

**Figure 9:**
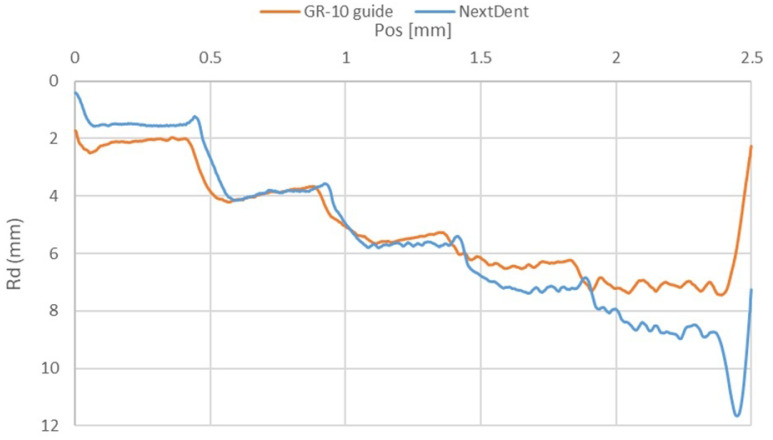
Residual scratch depth (Rd) on the surfaces of the tested materials.

In the scratch test, the permanent scratch depth (Rd) was determined, to assess the materials’ resistance to deformation under varying loads. While the overall depth of damage on the surfaces of the tested samples was similar (Figure 9), notable differences emerged under specific indenter forces. Under an indenter force of 1 N, a greater Rd was observed for the GR-10 guide material compared to NextDent SG. Conversely, at higher forces of 4 N and 5 N, higher Rd values were measured for the NextDent SG material.

This behavior can be explained by examining the materials’ hardness and deformation characteristics. Our hardness measurements revealed that NextDent SG possesses a higher hardness of approximately 118.664 MPa (HIT) and a higher modulus of elasticity of 2954.660 MPa (EIT), while GR-10 has a lower hardness of about 57.605 MPa and a modulus of elasticity of 1489.946 MPa. At the lower load of 1N, the softer and more compliant GR-10 material is more susceptible to plastic deformation, resulting in a greater residual depth. The higher hardness and stiffness of NextDent SG allow it to resist deformation more effectively under this load.

However, at higher loads of 4N and 5N, the deformation behavior shifts. The NextDent SG material, despite its higher hardness, may approach or exceed its yield point under increased stress, leading to significant plastic deformation and higher Rd values. In contrast, GR-10, although softer, may exhibit strain-hardening effects or possess greater toughness, enabling it to better withstand higher loads with less increase in residual depth. This suggests that under higher stresses, GR-10’s ability to distribute and absorb energy reduces the extent of deformation.

**Figure 10:**
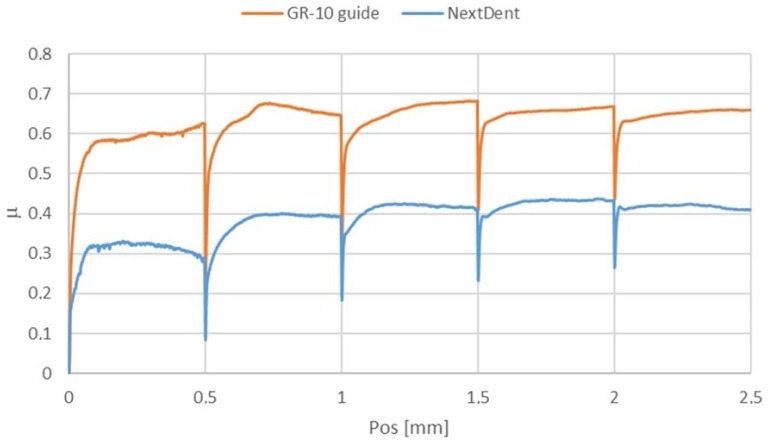
Coefficient of friction during the scratch test.

**Figure 11:**
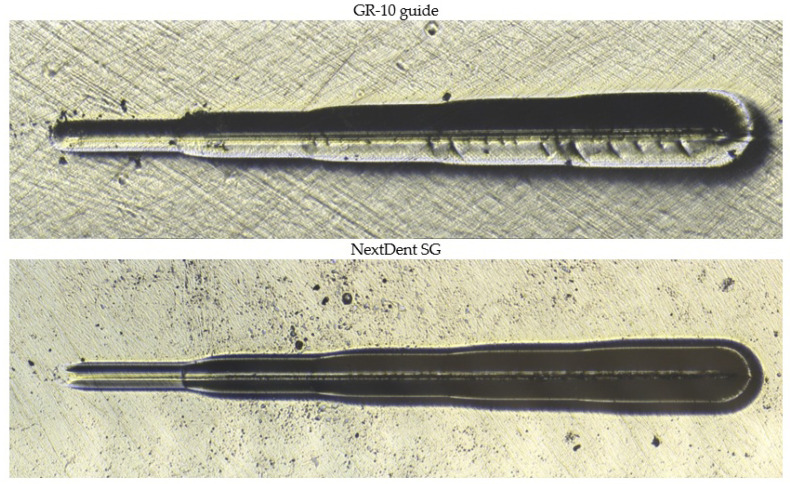
Optical microscope images of scratches on the surfaces of the tested materials.

Despite the similar permanent scratch depths, the coefficient of friction measured during scratching differed significantly between the materials (Figure 10). The GR-10 guide material demonstrated substantially higher resistance to scratching than NextDent SG. Microscopic observations (Figure 11) indicate that while the sizes of the scratches on both materials were similar, the NextDent SG samples exhibited pronounced “pile-up” [23] and cohesive damage. These features suggest different deformation mechanisms were at play, with NextDent SG undergoing more significant material displacement and surface damage.

### 3.4. Sliding Friction and Wear Results

**Figure 12:**
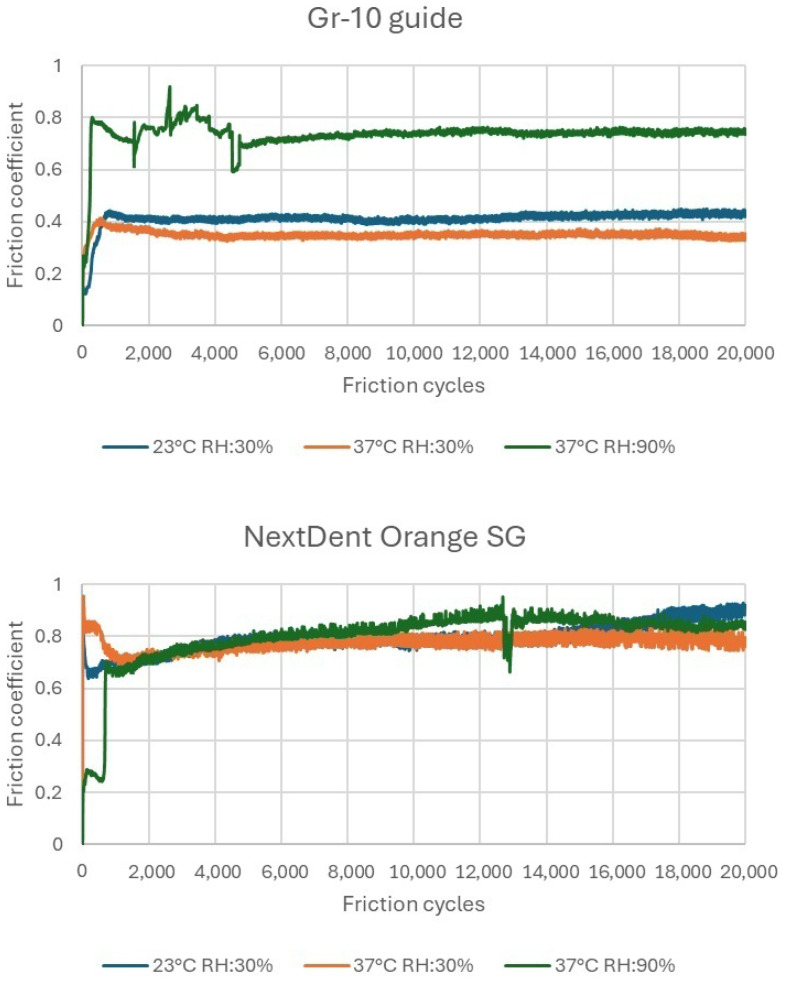
Mean of characteristics of the friction coefficient depending on the number of friction cycles.

The graphs (Figure 12) show the mean sliding friction coefficient depending on the number of friction cycles. In the case of the GR-10 guide material at 30% humidity, the changes in the friction coefficient were slight. Increasing the test temperature to 37 °C compared to room temperature caused a slight decrease in the friction coefficient. However, a significant increase in the friction coefficient was observed at 90% humidity and 37 °C. Under these conditions, the sample surface was covered with a visible water film.

Despite the presence of this water film, the friction coefficient did not decrease, as might be expected, due to improved lubrication. This counterintuitive behavior can be explained by the complex interactions between the water film and the GR-10 guide material’s surface properties. At high humidity and elevated temperatures, the water film may not act as an effective lubricant. Instead, it can enhance adhesive forces between the contacting surfaces through capillary action. The formation of capillary bridges increases the real area of contact and the normal force required to initiate and maintain sliding, resulting in a higher friction coefficient [24].

Additionally, the GR-10 guide material may absorb moisture under these conditions, leading to slight swelling or alterations in surface roughness. Increased roughness can amplify mechanical interlocking between surfaces, further elevating friction. The absorbed moisture might also plasticize the polymer matrix, reducing its hardness and causing greater deformation under load. These factors collectively contribute to the unexpected increase in the friction coefficient, demonstrating that the water film did not translate into improved lubrication for GR-10 guide [25].

The friction coefficient of the NextDent SG material changed differently. Regardless of the environmental conditions of the test, the friction coefficient value remained similar. Its value was approximately 0.8, which was higher than the value obtained for the GR-10 guide material.

**Figure 13:**
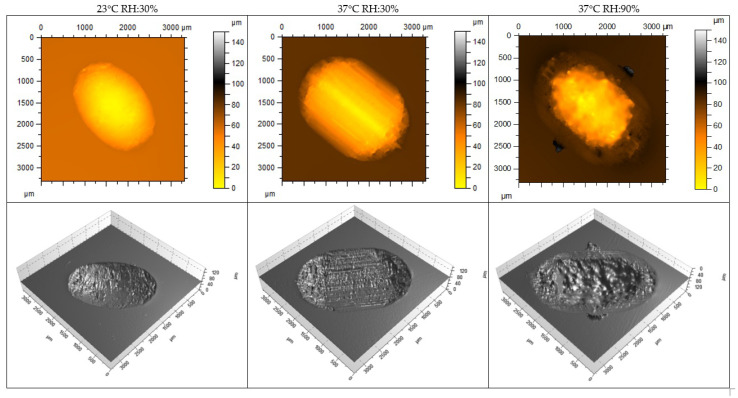
Wear measurement of the friction track GR-10 guide material specimens using a white light interferometry (WLI) microscope.

**Figure 14:**
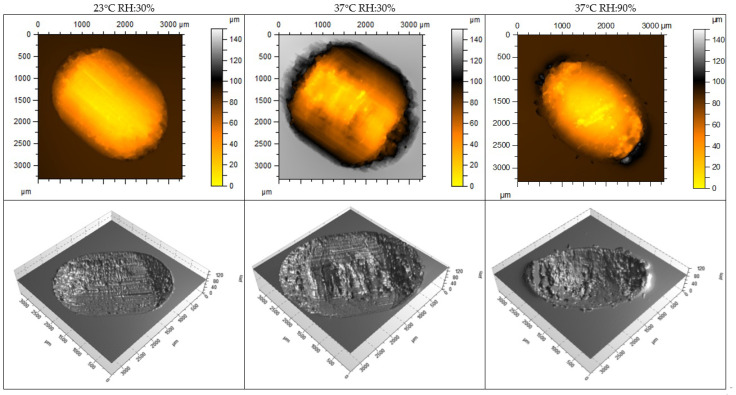
Wear measurement of the friction track NextDent SG material specimens using a white light interferometry (WLI) microscope.

Table 3 and Figure 13 and Figure 14 show the wear results, including measurement plots of the cross-sectional area, width, and depth of the friction track made using a white light interferometry (WLI) microscope from Taylor Hobson (Leicester, UK). The wear was varied. In the case of the material, the volumetric material loss after 20,000 friction cycles was the highest in the test, at 37 °C and 30% humidity. The highest volumetric wear of the NextDent SG material was measured under the same test conditions. The wear of the NextDent SG material was significantly higher. However, under test conditions of 37 °C and 90% humidity, the wear of both materials was the most similar. For the tested materials, there was no simple relationship between the friction coefficient and wear. It was noted that environmental conditions had a greater impact on wear than on the friction coefficient.

## 4. Discussion

Tribological processes are crucial in the physiological processes occurring in the oral cavity. Every person bite and grinds about 1 kg of food daily between their teeth. Tooth enamel wears out, but so do artificial dental materials [26]. There are also a number of other tribological phenomena in the oral cavity, including during orthodontic treatment [27,28]. Orthodontic devices are of particular importance in the treatment of orthodontic diseases. Disease processes affect the condition of orthodontic devices, due to biomechanical loads. Thegosis and bruxism can also result in friction and wear of orthodontic appliances. Thegosis is the action of sliding teeth into lateral positions [29]. This has been suggested to be a genetically determined habit originally established to sharpen teeth [29,30]. Bruxism is the action of grinding teeth without the presence of food, which is regarded as a response to stress and is treated clinically as pathological behavior [29,31]. The potential number of patients can be estimated. Researchers have found that over 5% of the population experiences clinical symptoms related to temporomandibular disorder (TMD) [32]. In other studies [33], it was found that up to 10% of the population may experience bruxism. Bruxism episodes occur at a frequency of 8.1 per hour [34]. In the presented friction and wear test results, the number of cycles corresponds to approximately 3 months of orthodontic splint use. Orthodontic splints are designed to take on pathological loads. The materials used to make them in the era of digital dentistry and 3D printing, mainly polymer materials, must be resistant to these conditions. Unfortunately, they sometimes get damaged [17]. In general, the wear of polymers depends on the hardness of the materials in the friction node, and operational wear is caused by micro-cutting, scratching, and grooving [35]. Despite wearing out, polymers are a very attractive group of materials for tribological systems, where they are often used in pairs with harder materials [36,37]. Special tribological cases include the interaction of human teeth with polymer orthodontic devices [38,39]. This system also occurs in the subject studies because the hardness of tooth tissues is much greater than the hardness of the tested polymer materials, which was also confirmed in the studies included in [4]. For comparison, the hardness of enamel is about 2750 MPa [40], and the hardness of the tested materials ranged from several tens to over a hundred megapascals (Table 2). In polymer materials, surface properties are not related to volumetric properties [41,42]. What is more, they change with operation time. In our studies of periodic indenter loading, a stepwise increase in the modulus of elasticity was observed, including a rapid increase in the modulus in the initial cycles, followed by a slow increase and stabilization. A similar relationship, but inversely proportional to the number of cycles, was observed in the case of indentation hardness. The initial and residual hardness of the tested orthodontic materials differed significantly. In applications where friction occurs between a polymer element and a much harder one, the hardness of the polymer plays an important role [43,44]. According to the specialized literature [43], hardness is particularly important for structures where contact stresses occur. It is very important for orthodontic device materials [45]. Hardness can also translate into tribological resistance [46,47]. In assessing tribological resistance, some researchers use the H/E ratio [48] (indentation hardness HIT to surface modulus of elasticity EIT). This ratio combines the ability to deform elastically as much as possible (low modulus) and the ability to minimize permanent deformation (high hardness). For the GR-10 guide material, this ratio was 0.33 in the first test cycle and 0.114 in the last, and for the NextDent SG material it was, respectively, 0.274 and 0.09. The higher the H/E ratio, the greater the wear resistance of the material should be [49]. For both materials, it decreased in the subsequent load cycles. NextDent SG had a lower H/E ratio in both cases, which is consistent with the results obtained in sliding friction tests. It has also been shown [50] that the hardness of many polymer materials is correlated with the coefficient of friction. In the presented studies, the GR-10 guide material, which was less hard, was characterized by a lower coefficient of friction, with the caveat that the combined effect of moisture and temperature close to physiological processes influenced the increase in the coefficient of friction. The coefficient of friction in hard material–polymer material systems in low-load tribological pairs is relatively low [51] and ranges from 0.05 to 0.6 [52]. In paper [53], it was reported that friction coefficient values between 0.4 and 0.5 are considered not small (but in the range typical for plastics). In the presented studies, a higher coefficient of friction was obtained in the predicted operating conditions. The scratch test has often been used to give a guide to the abrasive wear resistance of polymers [4,54,55]. In a clinical situation, scratches can be caused by sharp irregularities on the edge of the teeth; these irregularities can arise, among other reasons, as a result of enamel chipping on the outline of the incisal edge of the incisors in the disease process. Another cause of irregularities is the inaccurate preparation of the enamel and filling of the cavity [56]. This state of affairs contributes to the formation of irregularities on the outline line of the filling [18]. Scratching with a sharply ended element occurs as a result of the combined action of normal and tangential forces on the surface. Scratches can also be the result of the action of small particles/grains on the polymer surface [55,57,58]. Resistance to such damage is important from a practical point of view. Quantitatively, the degree of scratch resistance can also be related to the width, the depth of the scratch (Rd), or the volume of the resulting groove [59,60]. The NextDent SG material, which was more than twice as hard, was characterized by a higher Rd. The action of only the normal force, as in the hardness test, caused behavior different in this material from the behavior under the simultaneous action of normal and tangential forces (Pd). The tendency to groove on the surface of this material was also confirmed, based on SEM observations of the friction surface obtained in the cyclic sliding friction test. In a humid environment at physiological temperature, the mechanical properties of the tested materials deteriorate. The degree of change of the aging-dependent modulus of elasticity (values range from 0 to 1, where 0 means no loss of properties) in artificial saliva is 0.27 (GR-10 guide) and 0.11 (NextDent SG) [61]. The GR-10 guide material is characterized by a lower modulus of elasticity, which makes it more susceptible to deformation, and this may translate into a lower likelihood of causing tissue damage in the patient [62]. The impact of the synergistic effect of the humid environment and elevated temperature in the presented studies seems crucial. The application of J.S. Mill’s rule of eliminative induction [19] in the studies of oscillatory sliding friction and wear allowed for the assessment of the impact of isolated environmental factors and their synergistic effect on the friction and wear of the tested materials. The obtained research results are not obvious. Generally, wear at 37 °C was the highest, but increasing humidity at this temperature from 30% to 90% significantly reduced wear. This can be explained by the action of de-ionized water. Probably, moisture positively affected the reproducibility of the anti-wear surface layer. It is possible that the adoption of a frequency close to physiological and sinusoidal alternating stresses and strains did not disturb the water lubricating film. Water has a much lower viscosity than technical lubricating fluids. Water film is thin, sometimes less than 1 µm thick [63,64]. For most of the time, the oral cavity is exposed to a relatively constant-temperature humid environment. Some believe that water in the structure of a polymer matrix composite acts as a plasticizer and that its action leads to stress relaxation and reduced stiffness [25]. Cyclic sorption and desorption of water, in friction cycles on the friction surface, can cause internal mechanical stresses. It is believed that the process of water absorption into polymer materials is accelerated under cyclic fatigue loads [65]. The studies presented here confirm the importance of moisture for the tribological behavior of the tested materials. Moreover, they allow for a partial explanation of whether damage from mechanical and hydro-thermal loads adds up, i.e., whether the principle of superposition applies in the case of the combined action of these loads, or whether there is a phenomenon of synergy.

While the findings provide valuable insights into how humidity and temperature affect material performance, it is important to acknowledge that saliva plays a crucial role in the oral environment. Saliva’s chemical composition, enzymatic activity, and lubricating properties can significantly influence friction and wear behavior. Additionally, food particles can introduce abrasive interactions and alter the contact mechanics between the material surfaces and opposing dentition.

The process of water absorption into polymer materials is accelerated under cyclic fatigue loads [65]. The mechanism of hydrolytic degradation is not well understood. It is likely that chemical compounds in the aqueous solution cause the decomposition of the polymer network by breaking bonds [66]. Amorphous polymers are more susceptible to hydrolytic degradation than crystalline polymers, linear polymers more than branched ones and polymers with higher molecular weight. Degradation depends on the presence of specific chemical groups in the molecule, such as ester, amide, and urethane groups [67]. The intensity of degradation also depends on the surface condition of the material, the presence of defects, and the type and amount of additional substances (e.g., filler particles, initiators) [67,68]. The rate of water molecule diffusion decreases with the increasing hydrophilicity of the matrix, indicating that the interaction between resin and water molecules affects the diffusion rate [68,69,70].

Future research will aim to incorporate artificial saliva to create a more comprehensive simulation of oral conditions. By doing so, we can better understand the complex interplay of mechanical and chemical factors affecting the tribological performance of orthodontic materials.

## 5. Conclusions

Based on study of the specialized literature, research, and analyses, the following final conclusions were formulated:

1. Key Findings:
  (a) This study revealed that the mechanical and tribological properties of 3D-printed materials used in orthodontics, specifically NextDent SG and GR-10, are significantly affected by environmental conditions such as temperature and humidity.
  (b) Elevated temperature (37 °C) and high humidity (90%) were shown to have a substantial impact on the friction coefficients and wear behaviors of these materials.
  (c) Notably, the GR-10 material exhibited an unexpected increase in the friction coefficient under high humidity, which did not correlate with increased wear, indicating complex interactions between environmental factors and material properties.
2. Implications and Future Work:
  (a) The findings underscore the importance of conducting experimental evaluations to accurately assess the wear and performance of 3D-printed orthodontic materials.
  (b) Due to the limited number of studies and the diversity of available 3D printing technologies and materials, there is a pressing need for additional research in this area [71].
  (c) Future studies should focus on developing standardized testing methods to enable reliable comparisons across different materials and technologies.
3. Challenges in Comparative Analysis:
  (a) The ability to compare our results with those of other researchers was constrained by the scarcity of relevant studies and the significant variability in research methodologies.
  (b) This highlights the necessity for a more unified approach in testing and reporting, to advance the collective knowledge in this field.
4. Environmental Effects on Material Properties:
  (a) The pronounced impact of elevated temperature and humidity on the tribological properties of the tested materials suggests that in vivo conditions can considerably influence material performance.
  (b) The unexpected behavior of the GR-10 material under high humidity conditions warrants further investigation, to understand the underlying mechanisms.
5. Recommendations for Future Research:
  (a) Conducting cyclic tests is essential, as the properties of the tested materials were observed to change significantly after just a few dozen indentation cycles.
  (b) Long-term studies that simulate the actual oral environment over extended periods are recommended, to evaluate the durability and reliability of these materials in clinical applications.

## Figures and Tables

**Table 1:** Parameters characterizing the surface topography of the tested samples (average results for three measurements of samples of each material).

Parameter [µm]	NextDent SG Orange		GR-10 Guide	
Stan	*	**	*	**
Sq	6.615	0.020	4.113	0.034
Ssk	1.479	−0.532	0.943	−0.376
Sku	7.814	6.453	4.679	4.076
Sp	39.180	0.250	25.554	0.305
Sv	28.840	0.407	9.719	0.396
Sz	68.020	0.657	35.273	0.701
Sa	4.772	0.016	3.178	0.027

* before processing on the grinder-polisher; ** after polishing.

**Table 2:** Descriptive statistics of indentation hardness, indentation modulus, and stiffness of the surface of the tested materials.

Parameters	N (Number of Measurements) = 15 (3 × 5), n (Number of Samples) = 3	Gr 10 Guide	NextDent SG
HIT [MPa]	Mean	57.605	118.664
	Std Dev	5.025	6.640
	Min	51.804	111.854
	Max	69.489	136.399
EIT [MPa]	Mean	1489.947	2954.660
	Std Dev	140.118	93.034
	Min	1288.765	2796.381
	Max	1725.304	3152.841
S [N/µm]	Mean	0.256	0.352
	Std Dev	0.026	0.008
	Min	0.201	0.342
	Max	0.310	0.369

**Table 3:** Mean wear on the surface of the tested materials.

Material		GR-10 Guide			NextDent SG	
Temperature	23 °C RH:30%	37 °C RH:30%	37 °C RH:90%	23 °C RH:30%	37 °C RH:30%	37 °C RH:90%
Maximum depth [μm]	58.84	82.16	88.218	88.518	131.354	87.895
Volume [${\mathrm{mm}}^{3}$]	0.081	0.175	0.128	0.221	0.440	0.161
Cross section area [${\mathrm{mm}}^{2}$]	3.758	5.169	5.047	6.770	8.864	4.658

## Data Availability

The original contributions presented in this study are included in the article. Further inquiries can be directed to the corresponding authors.

## References

[1] Mayer J., Kessler A., Wöstmann B., Ernst C.-P. (2021). Influence of Cleaning Methods after 3D Printing on Two-Body Wear and Fracture Load of Resin-Based Temporary Crown and Bridge Material. Clin. Oral Investig..

[2] Kessler A., Hedderich J., Ernst C.-P., Hassel A.J. (2019). Three-Body Wear of 3D Printed Temporary Materials. Dent. Mater..

[3] Tahayeri A., Morgan M.C., Fugolin A.P.P., Bompolaki D., Athirasala A., Pfeifer C.S., Ferracane J.L., Bertassoni L.E. (2018). 3D Printed versus Conventionally Cured Provisional Crown and Bridge Dental Materials. Dent. Mater..

[4] Firlej M., Pieniak D., Niewczas A.M., Walczak A., Domagała I., Borucka A., Przystupa K., Igielska-Kalwat J., Jarosz W., Biedziak B. (2021). Effect of Artificial Aging on Mechanical and Tribological Properties of CAD/CAM Composite Materials Used in Dentistry. Materials.

[5] Domagała I., Przystupa K., Firlej M., Pieniak D., Niewczas A., Biedziak B. (2020). Bending Behaviour of Polymeric Materials Used on Biomechanics Orthodontic Appliances. Materials.

[6] Kroma A., Adamczak O., Sika R., Górski F., Kuczko W., Grześkowiak K. (2020). Modern Reverse Engineering Methods Used to Modification of Jewelry. Adv. Sci. Technol. Res. J..

[7] Jabłońska M., Jurczak W., Ozimina D., Adamiak M. (2023). Increasing the Operational Reliability of a Ship by Using a Composite Impeller in the Event of Hydrophore Pump Failure. Eksploat. Niezawodn. Maint. Reliab..

[8] Prause E., Hey J., Beuer F., Schmidt F. (2022). Wear Resistance of 3D-Printed Materials: A Systematic Review. Dent. Rev..

[9] Gómez-Mascaraque L.G., Aguilar M.R., San Román J., Aguilar M.R., San Román J. (2014). The Use of Smart Polymers in Medical Devices for Minimally Invasive Surgery, Diagnosis, and Other Applications. Smart Polymers and Their Applications.

[10] Kilikevičius A., Maciūnas K., Strazdaitė V., Niaura G., Rutkūnas V., Ostrauskaite J. (2018). Modelling of Silk-Reinforced PDMS Properties for Soft Tissue Engineering Applications. Technol. Health Care.

[11] Lamprou D.A., Narayan R., Narayan R. (2019). Polymeric Coatings and Their Fabrication for Medical Devices. Encyclopedia of Biomedical Engineering.

[12] Chopra A.M., Reddy A.S., Muliya V.S., Weisz G., Ternus B., Khheria B.K. (2017). Polymer Coating Embolism from Intravascular Medical Devices: A Clinical Literature Review. Cardiovasc. Pathol..

[13] Piedra-Cascón W., Álvarez-Quintana J., López-Álvarez M., Rodríguez-Urrego F., Martínez-González J. (2021). 3D Printing Parameters, Supporting Structures, Slicing, and Postprocessing Procedures of Vat-Polymerization Additive Manufacturing Technologies: A Narrative Review. J. Dent..

[14] (2015). Additive Manufacturing—General Principles—Part 2: Overview of Process Categories and Feedstock.

[15] Bagheri A., Jin J. (2019). Photopolymerization in 3D Printing. ACS Appl. Polym. Mater..

[16] Dikova T.D., Dzhendov D.A., Ivanov D., Bliznakova K. (2018). Dimensional Accuracy and Surface Roughness of Polymeric Dental Bridges Produced by Different 3D Printing Processes. Arch. Mater. Sci. Eng..

[17] Hirai K., Ikawa T., Shigeta Y., Shigemoto S., Ogawa T. (2017). Evaluation of Sleep Bruxism with a Novel Designed Occlusal Splint. J. Prosthodont. Res..

[18] Jańczuk Z. (2008). Stomatologia Zachowawcza: Zarys Kliniczny.

[19] Luty W. (1997). Badania Empiryczne. Wybrane Zagadnienia Metodyczne.

[20] (2012). Geometrical Product Specifications (GPS)—Surface Texture: Areal.

[21] Oliver W.C., Pharr G.M. (2004). Measurement of Hardness and Elastic Modulus by Instrumented Indentation: Advances in Understanding and Refinements to Methodology. J. Mater. Res..

[22] Sneddon I.N. (1965). The Relation between Load and Penetration in the Axisymmetric Boussinesq Problem for a Punch of Arbitrary Profile. Int. J. Eng. Sci..

[23] Hardiman M., Vaughan T.J., McCarthy C.T. (2016). The Effects of Pile-Up, Viscoelasticity and Hydrostatic Stress on Polymer Matrix Nanoindentation. Polym. Test..

[24] Maeda N., Chen N., Tirrell M., Israelachvili J.N. (2002). Adhesion and Friction Mechanisms of Polymer-on-Polymer Surfaces. Science.

[25] Ferracane J.L. (2006). Hygroscopic and Hydrolytic Effects in Dental Polymer Networks. Dent. Mater..

[26] Hebda M. (2007). Procesy Tarcia, Smarowania i Zużywania Maszyn.

[27] Barbosa L.M., da Silva W.M., de Mello J.D.B. (2019). Orthomicrotribometer. Wear.

[28] Zhang R., Han B., Liu X. (2023). Functional Surface Coatings on Orthodontic Appliances: Reviews of Friction Reduction, Antibacterial Properties, and Corrosion Resistance. Int. J. Mol. Sci..

[29] Zhou Z., Zheng J. (2008). Tribology of Dental Materials: A Review. J. Phys. D Appl. Phys..

[30] Every R.G., Kühne W.G., Kermack D.M., Kermack K.A. (1971). Biomodal Wear of Mammalian Teeth. Early Mammals.

[31] Mair L.H., Duxbury A.J., Dorr P.K., Beech I.D., Gilmour W.J., Jones S.S. (1996). Wear: Mechanisms, Manifestations and Measurement. Report of a Workshop. J. Dent..

[32] Peterson L.J. (2012). Peterson’s Principles of Oral and Maxillofacial Surgery.

[33] Ferendiuk E., Zajdel K., Pihut M. (2014). Incidence of Otolaryngological Symptoms in Patients with Temporomandibular Joint Dysfunctions. Biomed Res. Int..

[34] Beddis H., Pemberton M., Davies S. (2018). Sleep Bruxism: An Overview for Clinicians. Br. Dent. J..

[35] Wieleba W. (2013). Bezobsługowe Łożyska Ślizgowe z Polimerów Termoplastycznych.

[36] Slapnik J., Stiller T., Wilhelm T., Hausberger A. (2020). Influence of Solid Lubricants on the Tribological Performance of Photocurable Resins for Vat Photopolymerization. Lubricants.

[37] Chernets M. (2019). Method of Calculation of Tribotechnical Characteristics of the Metal-Polymer Gear, Reinforced with Glass Fiber, Taking into Account the Correction of Tooth. Eksploat. Niezawodn. Maint. Reliab..

[38] Mangal U., Min Y.J., Seo J.-Y., Kim D.-E., Cha J.-Y., Lee K.-J., Kwon J.-S., Choi S.-H. (2020). Changes in Tribological and Antibacterial Properties of Poly(Methyl Methacrylate)-Based 3D-Printed Intraoral Appliances by Incorporating Nanodiamonds. J. Mech. Behav. Biomed. Mater..

[39] Albo Hassan A.F., Jameel A., Nahidh M., Hamid D. (2019). Wear Resistance of Occlusal Splint Materials. J. Stomatol..

[40] Chun K.J., Choi H.H., Lee J.Y. (2014). Comparison of Mechanical Property and Role between Enamel and Dentin in the Human Teeth. J. Dent. Biomech..

[41] Niewczas A.M., Pieniak D., Ogrodnik P. (2012). Reliability Analysis of Strength of Dental Composites Subjected to Different Photopolymerization Procedures. Eksploat. Niezawodn. Maint. Reliab..

[42] Pieniak D., Niewczas A., Kordos P. (2012). Influence of Thermal Fatigue and Ageing on the Microhardness of Polymer-Ceramic Composites for Biomedical Applications. Eksploat. Niezawodn. Maint. Reliab..

[43] Dulebová Ľ., Garbacz T. (2017). The Effect of Particulate Fillers on Hardness of Polymer Composite. Adv. Sci. Technol. Res. J..

[44] Świderski A., Borucka A., Jacyna-Gołda I., Szczepański E. (2019). Wear of Brake System Components in Various Operating Conditions of Vehicle in the Transport Company. Eksploat. Niezawodn. Maint. Reliab..

[45] Nowakowska-Toporowska A., Małecka K., Raszewski Z., Wieckiewicz W. (2019). Changes in Hardness of Addition-Polymerizing Silicone-Resilient Denture Liners after Storage in Artificial Saliva. J. Prosthet. Dent..

[46] Mystkowska J., Dąbrowski J.R. (2010). Tribological Characteristics of the Kinematic Couple: Tooth–Composite Material for Permanent Dental Fillings. Eksploat. Niezawodn. Maint. Reliab..

[47] Pieniak D., Walczak A., Walczak M., Przystupa K., Niewczas A.M. (2020). Hardness and Wear Resistance of Dental Biomedical Nanomaterials in a Humid Environment with Non-Stationary Temperatures. Materials.

[48] Matthews A., Leyland A. (2006). The Role of Nanocomposite Coatings in Surface Engineering. Surface Engineering—Proceedings of the 5th International Surface Engineering Conference.

[49] Łępicka M., Grądzka-Dahlke M., Pieniak D., Pasierbiewicz K., Kryńska K., Niewczas A. (2019). Tribological Performance of Titanium Nitride Coatings: A Comparative Study on TiN-Coated Stainless Steel and Titanium Alloy. Wear.

[50] Myshkin N.K., Petrokovets M.I., Kovalev A.V. (2005). Tribology of Polymers: Adhesion, Friction, Wear, and Mass-Transfer. Tribol. Int..

[51] Krzyżak A., Racinowski D., Szczepaniak R., Kosicka E. (2023). An Assessment of the Reliability of CFRP Composites Used in Nodes of Friction after Impact of UV-A Impacts and Thermal Shocks. Eksploat. Niezawodn. Maint. Reliab..

[52] Bhushan B. (2013). Principles and Applications of Tribology.

[53] Hanon M.M., Ghaly A., Zsidai L., Klébert S. (2022). Tribological Characteristics of Digital Light Processing (DLP) 3D Printed Graphene/Resin Composite: Influence of Graphene Presence and Process Settings. Mater. Des..

[54] Abdelbary A., Abdelbary A. (2014). Wear of Polymer Composites. Wear of Polymers and Composites.

[55] Pieniak D., Kubiak A., Firlej M., Pieniak K., Niewczas A.M. (2019). Testing Methods for Determining the Scratch Resistance of Firefighter’s Helmets. Przemysł Chem..

[56] Niewczas A., Pieniak D., Bachanek T., Surowska B., Bieniaś J., Pałka K. (2010). Prognosing of Functional Degradation of Bio-Mechanical Systems Exemplified by the Tooth-Composite Filling System. Eksploat. Niezawodn. Maint. Reliab..

[57] Chen Z., Wu L.Y., Friedrich K., Schlarb A.K. (2013). Tribology of Polymeric Nanocomposites. Tribology of Polymeric Nanocomposites.

[58] Bora M.O., Coban O., Sinmazçelik T. (2010). Mechanical Properties and Performance of Polymer Matrix Composites. J. Reinf. Plast. Compos..

[59] Palaniappan S., Celis J.P., Van Meerbeek B., Peumans M., Lambrechts P. (2013). Correlating In Vitro Scratch Test with In Vivo Contact Free Occlusal Area Wear of Contemporary Dental Composites. Dent. Mater..

[60] Andena L., Chiarot G. (2022). Scratch Hardness as a Quasi-Intrinsic Parameter to Measure the Scratch Resistance of Polymers. Wear.

[61] Firlej M., Pieniak D., Kubiak A., Niewczas A., Pieniak K. (2021). Analysis of Indentation Hardness, Scratch Resistance, and Sliding Wear of Polymeric Materials from 3D DLP UV Resin. Przemysł Chem..

[62] Potewiratnanond P., Ekrojanakul C., Harikul T. (2023). Wear Effects between Polymethyl Methacrylate Occlusal Splints and Opposing Dentin Surfaces during Bruxism Mimicking Events. BDJ Open.

[63] Olszewski A., Ozimina D. (2013). Łożyska Ślizgowe Smarowane Wodą. Eksploatacja Systemów Tribologicznych.

[64] Tropp M., Lukáč M., Benko M., Brumerčík F., Krzysiak Z., Nieoczym A. (2020). Sealing Technology for Vacuum Applications Working by Increased Temperatures. Advances in Mechanisms Design.

[65] Lohbauer U., Belli R., Ferracane J.L. (2013). Factors Involved in Mechanical Fatigue Degradation of Dental Resin Composites. J. Dent. Res..

[66] Finer Y., Santerre J.P. (2004). The Influence of Resin Chemistry on a Dental Composite’s Biodegradation. J. Biomed. Mater. Res. Part A.

[67] Ammar-Khodja I., Picard C., Fois M., Marais C., Netchitailo P. (2009). Preliminary Results on Thermo-Oxidative Ageing of Multi-Hole Carbon/Epoxy Composites. Compos. Sci. Technol..

[68] Sobków D., Barton J., Czaja K., Sudoł M., Mazoń B. (2014). Studies on the Resistance of Materials to the Impact of Natural Environmental Factors. Chemik.

[69] Merdas I., Thominette F., Tcharkhtchi A., Verdu J. (2002). Factors Governing Water Absorption by Composite Matrices. Compos. Sci. Technol..

[70] Xian G., Bai Y., Qi X., Wang J., Tian J., Xiao H. (2021). Hygrothermal Aging on the Mechanical Property and Degradation Mechanism of Carbon Fiber Reinforced Epoxy Composites Modified by Nylon 6. J. Mater. Res. Technol..

[71] Rouf S., Raina A., Haq M.I.U., Naveed N., Jeganmohan S., Kichloo A.F. (2022). 3D Printed Parts and Mechanical Properties: Influencing Parameters, Sustainability Aspects, Global Market Scenario, Challenges, and Applications. Adv. Ind. Eng. Polym. Res..

